# Experience in Prehospital Endotracheal Intubation Significantly Influences Mortality of Patients with Severe Traumatic Brain Injury: A Systematic Review and Meta-Analysis

**DOI:** 10.1371/journal.pone.0141034

**Published:** 2015-10-23

**Authors:** Sebastiaan M. Bossers, Lothar A. Schwarte, Stephan A. Loer, Jos W. R. Twisk, Christa Boer, Patrick Schober

**Affiliations:** 1 Department of Anaesthesiology, VU University Medical Centre, Amsterdam, The Netherlands; 2 Helicopter Emergency Medical Service "Lifeliner 1", VU University Medical Centre, Amsterdam, The Netherlands; 3 Department of Epidemiology and Biostatistics, VU University Medical Centre, Amsterdam, The Netherlands; 4 Institute for Cardiovascular Research, VU University Medical Centre, Amsterdam, The Netherlands; University of Florida, UNITED STATES

## Abstract

**Background:**

Patients with severe traumatic brain injury (TBI) are at high risk for airway obstruction and hypoxia at the accident scene, and routine prehospital endotracheal intubation has been widely advocated. However, the effects on outcome are unclear. We therefore aim to determine effects of prehospital intubation on mortality and hypothesize that such effects may depend on the emergency medical service providers’ skill and experience in performing this intervention.

**Methods and Findings:**

PubMed, Embase and Web of Science were searched without restrictions up to July 2015. Studies comparing effects of prehospital intubation versus non-invasive airway management on mortality in non-paediatric patients with severe TBI were selected for the systematic review. Results were pooled across a subset of studies that met predefined quality criteria. Random effects meta-analysis, stratified by experience, was used to obtain pooled estimates of the effect of prehospital intubation on mortality. Meta-regression was used to formally assess differences between experience groups. Mortality was the main outcome measure, and odds ratios refer to the odds of mortality in patients undergoing prehospital intubation versus odds of mortality in patients who are not intubated in the field. The study was registered at the International Prospective Register of Systematic Reviews (PROSPERO) with number CRD42014015506. The search provided 733 studies, of which 6 studies including data from 4772 patients met inclusion and quality criteria for the meta-analysis. Prehospital intubation by providers with limited experience was associated with an approximately twofold increase in the odds of mortality (OR 2.33, 95% CI 1.61 to 3.38, p<0.001). In contrast, there was no evidence for higher mortality in patients who were intubated by providers with extended level of training (OR 0.75, 95% CI 0.52 to 1.08, p = 0.126). Meta-regression confirmed that experience is a significant predictor of mortality (p = 0.009).

**Conclusions:**

Effects of prehospital endotracheal intubation depend on the experience of prehospital healthcare providers. Intubation by paramedics who are not well skilled to do so markedly increases mortality, suggesting that routine prehospital intubation of TBI patients should be abandoned in emergency medical services in which providers do not have ample training, skill and experience in performing this intervention.

## Introduction

Severe traumatic brain injury (TBI) is a leading cause of death and morbidity in the first decades of life, with a tremendous burden on the society due to high costs of care and loss of productive life years [1–7]. Prehospital emergency care is the first step in the chain of survival, and effective treatment during this period is considered crucial for a beneficial outcome [8]. However, current prehospital TBI-treatment guidelines are based on low quality of evidence [9], and optimal treatment is a matter of on-going debate.

Airway obstruction is common at the accident scene in patients with severe TBI [10, 11], and resulting hypoxaemia and hypercapnia are known to trigger secondary injuries that adversely affect outcome [12, 13]. While isolated TBI *per se* does not necessarily lead to airway obstruction, a depressed level of consciousness associated with TBI may lead to airway obstruction due to displacement of the epiglottis, tongue or soft palate [14, 15]. Unconsciousness is also associated with compromised protective airway reflexes, which put the patient at increased risk of aspiration of gastric contents and blood [16, 17], especially when oropharyngeal bleeding is present. Additionally, head injury can induce apnoea [18], and other concomitant injuries such as chest trauma can also contribute to hypoxia [19].

For these reasons, securing the airway is considered a first treatment priority, and prehospital endotracheal intubation–as the "gold standard" of airway management–has often been advocated for comatose trauma patients with a Glasgow Coma Scale (GCS) score of ≤ 8 [20, 21]. However, despite theoretical advantages and despite widespread use by emergency medical services (EMS) around the world, there is little scientific evidence to support this practice [22]. In fact, it has even been suggested that prehospital endotracheal intubation may be associated with increased mortality [23]. Hypoxia due to prolonged or failed intubation attempts, increases in intracranial pressure during laryngoscopy, haemodynamic effects of drugs used to facilitate intubation, as well as inappropriate ventilation after endotracheal intubation might all contribute to unfavourable outcomes. In many paramedic based EMS systems, prehospital endotracheal intubation is performed by paramedics who only have basic training in this procedure and infrequently perform intubations in clinical practice. On the other hand, in other EMS systems, endotracheal intubation may be performed by highly trained critical care personnel or emergency physicians. In this context, it is likely that the incidence of adverse events is associated with the level of training and experience in airway management of the provider who performs prehospital intubation (PHI). We therefore hypothesize that PHI by highly trained providers is beneficial, while the same intervention performed by less skilled personnel may be detrimental. We systematically reviewed the available literature and performed a stratified meta-analysis and meta-regression of eligible studies to assess effects of PHI on mortality in patients with severe TBI in the context of the EMS-providers’ experience.

## Methods

### Protocol and registration

This study was conducted in accordance with PRISMA (Preferred Reporting Items for Systematic Reviews and Meta-Analyses) [24, 25] and MOOSE (Meta-Analysis of Observational Studies in Epidemiology) [26] guidelines. The search strategy, study selection, bias assessment, as well as data extraction and analysis techniques were specified a priori. The study was registered at the International Prospective Register of Systematic Reviews (PROSPERO) with number CRD42014015506 (http://www.crd.york.ac.uk/PROSPERO/display_record.asp?ID=CRD42014015506).

### Eligibility criteria

Articles of interest were fully published controlled trials and observational studies comparing PHI versus non-invasive prehospital airway management in patients with suspected or confirmed severe TBI. Severe TBI was defined as a prehospital/admission Glasgow Coma Scale (GCS) ≤ 9 in the presence of a trauma mechanism or findings at physical examination suggestive of head injury, or a Head Abbreviated Injury Score (H-AIS) ≥ 3. Outcome of interest was mortality, and studies that reported or allowed calculation of an effect size were selected. Studies specifically investigating paediatric patients were excluded. Manuscripts reporting other patient populations were considered eligible as long as the data relating to the TBI population could be extracted.

A subset of studies was selected for the meta-analysis and meta-regression. Selection criteria were: (1) sufficient quality as described in detail below; (2) the overall study-level EMS-provider experience could be determined; and (3) mortality could be meaningfully compared between intubated and non-intubated patients, i.e., groups were drawn from the same population and are either directly comparable by design with respect to baseline characteristics and injury severity, or adjusted analyses were used to address imbalances between both cohorts.

When multiple publications with overlapping data met eligibility criteria for the meta-analysis, or when the same manuscript reported multiple eligible analyses of overlapping data, we used only one of the analyses to avoid duplicate inclusion of patients. In this case, the analysis in which the effect size was estimated with highest precision (i.e., with smallest standard error) was selected.

### Information sources and search strategy

We searched PubMed, Embase and Web of Science, without any restrictions, to identify eligible publications. This search was last updated on July 11^th^, 2015. For PubMed, the following search strategy was used: *("intubation*, *intratracheal"[Mesh] OR "intubation"[Mesh] OR "intubation"[All Fields]) AND ("brain injuries"[Mesh] OR "brain injuries"[All Fields] OR ("brain"[All Fields] AND "injuries"[All Fields]) OR ("head"[All Fields] AND ("injuries"[All Fields] OR "trauma"[All Fields])) OR ("traumatic"[All Fields] AND "brain"[All Fields] AND "injury"[All Fields]) OR ("traumatic brain injury"[All Fields]) OR ("head injury"[All Fields]) OR ("head trauma"[All Fields])) AND ("emergency medical services"[Mesh] OR "prehospital"[All Fields])*. The search terms were adapted accordingly for the other databases. Reference lists of pertinent publications were also screened for eligible studies.

### Study selection

Two investigators (SMB, PS) independently assessed publications for eligibility by screening abstracts of all identified studies. Full text articles were retrieved for all publications for which the relevance could not be determined based on title and abstract. Disagreements on eligibility were discussed among the investigators, and a third investigator (LAS) was appointed to resolve persisting disagreements.

### Data extraction

Data were extracted by one author (PS) using a standardized data collection sheet, and all data were checked for completeness and accuracy by a second author (SMB). We abstracted information from each included study on: (1) study characteristics, including design, population size, inclusion and exclusion criteria as well as time period and geographical area of patient inclusion; (2) patient characteristics, including age, gender and injury severity; (3) treatments in the intervention and control group; and (4) outcome measures. Studies were classified according to the level of training and experience in performing endotracheal intubations of the group of providers delivering prehospital care. Studies were labelled as “limited experience” if intubation was performed by personnel who usually have basic skills in this technique and who commonly only infrequently perform intubations in routine practice (e.g., emergency medical technicians and paramedics with limited scope of practice). “Extended experience” was selected if intubation was performed by prehospital emergency physicians or nurses/paramedics with an extended scope of practice and training (e.g., specially trained critical care paramedics/nurses). Studies in which the patient population was intubated by a heterogeneous group of providers or in which the level of training could not be ascertained were classified as “indeterminate”. Three reviewers (SMB, LAS, PS) independently assessed and scored the level of experience, and a level was only assigned by unanimous consensus.

Seven authors were contacted to obtain additional information, however only three responses were received.

### Assessment of study quality and risk of bias within studies

Quality assessment was independently performed by two authors (SMB and PS), and a third author (LAS) was consulted in case of disagreement. We used the Newcastle-Ottawa scale to assess the risk of bias of cohort studies [27]. A total of nine stars could be allocated per study for selection of participants, comparability of study groups and assessment of outcome. A total score of ≥ 7 stars with full score for "comparability" were required as eligibility for the meta-analysis.

Randomized controlled trials (RCT) were scored using the Cochrane Collaboration’s tool for assessing risk of bias [28]. This tool is used to classify studies as "low", "unclear" and "high" risk of bias. Since blinding of EMS-providers and patients is not possible in studies comparing PHI versus other airway management, this respective item was omitted and studies were classified as "low" risk of bias if no other sources of bias could be identified. Trials with low risk of bias were considered eligible for the meta-analysis.

### Data synthesis and statistical analysis

The primary measure of the treatment effect was the odds ratio (OR) of mortality in patients undergoing PHI versus patients who were not intubated in the prehospital setting. No attempt was made to meta-analyse data across studies that did not meet the described criteria; these publications are only presented descriptively. A meta-analysis of eligible data was performed with STATA 13.0 (StataCorp, Texas). In accordance with our hypothesis that the effect of PHI on mortality differs with EMS-provider experience, and to accommodate for other potential between study heterogeneity, we used a random effects model [29]. Additionally, the analysis was stratified on the EMS-provider's level of experience. Heterogeneity was quantified as the percentage of total variation across studies that is attributable to heterogeneity rather than chance (I^2^-statistic) [30]. Random effects meta-regression with EMS-provider experience as trial-level covariate was used to formally assess differences between groups of EMS-providers [31].

### Assessment risk of bias between studies

We addressed small-study bias in the meta-analysis by plotting the natural logarithm of the odds ratio against its standard error. Funnel plot asymmetry was assessed by Egger’s regression asymmetry test [32].

## Results

### Study selection

**Fig 1:**
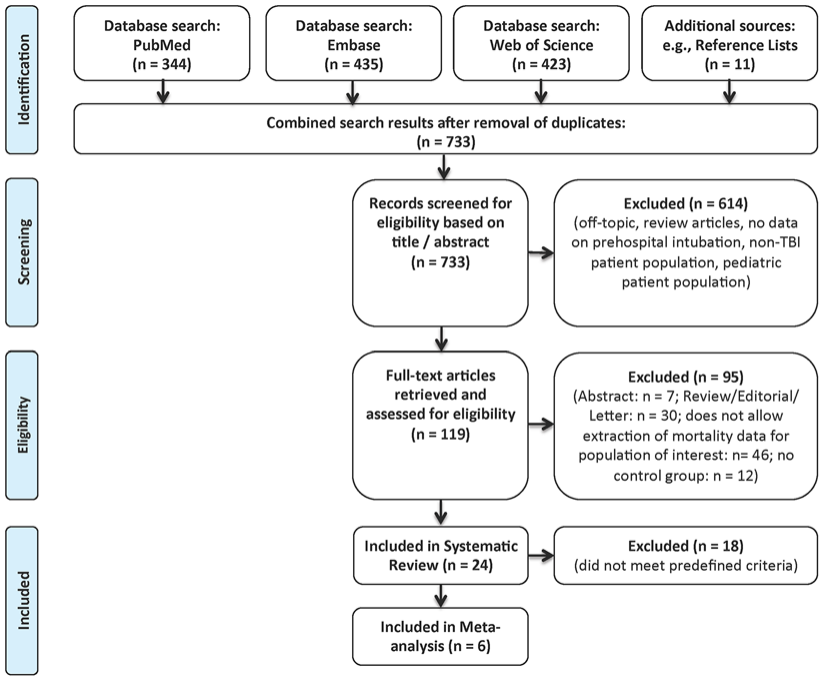
PRISMA flow diagram. PRISMA flow diagram summarizing identification, screening, eligibility and inclusion of studies.

The database search provided a total of 1202 articles. Screening of reference lists identified 11 additional articles. A total of 733 articles remained after removal of duplicates/triplicates. Based on review of the abstracts, 614 papers failed to meet the inclusion criteria. The full text of the remaining 119 articles was retrieved and assessed for eligibility. Of those, 95 articles were discarded because they did not meet the inclusion criteria. The remaining 24 studies were included in the systematic review, and six of those studies met the inclusion criteria for the meta-analysis. See Fig 1 for the PRISMA flow diagram.

### Study characteristics

Twenty-four studies reporting data from more than 30,000 patients were selected for the systematic review [33–56]. Due to partial geographic and temporal overlap of some of the studies (Table 1), the exact number of included individual patients could not be determined. Eighteen studies were performed in North America, four in Europe, one in Australia and one in Southwest Asia.

**Table 1:** Study characteristics.

First author (year)	Study design	Study Period	Region	Inclusion criteria^a^	Exclusion criteria^a^	n total^b^
Bernard (2010) [33]	Randomized controlled trial	2004–2008	Victoria, Australia	Age ≥ 15 years, evidence for head trauma, GCS ≤ 9, intact airway reflexes	Within 10 minutes of trauma hospital, no intravenous access, allergy to RSI drugs, transport planned by helicopter	312
Bochicchio (2003) [34]	Prospective cohort study	2000–2001	Maryland, USA	Adult trauma patients with GCS ≤ 8 and H-AIS ≥ 3	Death within 48h of admission, failed intubation in the field (>2 attempts), long field extrications, transfer from outside institutions	191
Bukur (2011) [35]	Retrospective cohort study	2005–2009	Los Angeles County, USA	Age ≥ 14 years, H-AIS ≥ 3 and all other AIS < 3, intubation required either in the pre-hospital period or in the ED	Dead on arrival or in emergency room, non-survivable injuries (any AIS = 6), missing intubation data	2366^c^
Davis (2003) [37]	Matched cohort study, prospectively enrolled intervention group matched to historical controls	Intervention: 1998–2000; Controls: "past 10 years, preference given to patients in past 5 years"	San Diego County, USA	Intervention: Apparent age ≥ 18, major trauma criteria (per county protocol) with suspected head injury, GCS ≤ 8, estimated transport time to ED > 10 minutes, intubation without RSI medication unsuccessful or impossible	Intervention: inability to obtain iv-access, violation of RSI protocol, CPR before administration of RSI drugs, inability to be intubated by prehospital personnel, transport to non-trauma centre, H-AIS < 2 or higher H-AIS defined by neck injury, death in the field or ED within 30 minutes	836^d^
Davis (2004) [36]	Matched cohort study, prospectively enrolled intervention group matched to historical controls	Intervention: 1998–2002; Controls: NR	San Diego County, USA	See Davis (2003)	See Davis (2003) + incomplete oximeter-capnometer data	236^d^
Davis (2005a) [39]	Retrospective cohort study	1987–2003	San Diego County, USA	Major trauma with GCS ≤ 8 and H-AIS ≥ 3	H-AIS defined by non-head injury, incomplete data, interfacility transport	2243^d^
Davis (2005b) [40]	Retrospective cohort study	1987–2003	San Diego County, USA	Major trauma with H-AIS ≥ 3, sub-analyses reported for patients with GCS ≤ 8 and/or H-AIS ≥ 4	H-AIS defined by neck injury	2474 to 9503^d^
Davis (2005c) [41]	Matched cohort study, prospectively enrolled intervention group matched to historical controls	Intervention: 1998–2002; Controls: NR	San Diego County, USA	See Davis (2003)	See Davis (2003)	1056^d^
Davis (2006) [38]	Retrospective cohort study	1992–2003	San Diego County, USA	Adult major trauma victims with H-AIS ≥ 3	H-AIS defined by non-head injury, CPR in the field, missing arrival ABG data	3804^d^
Franschman (2011) [42]	Retrospective cohort study	2003–2007	Amsterdam and Nijmegen region, the Netherlands	Age ≥ 16 years, CT confirmed TBI and GCS ≤ 8 primarily admitted to one of two participating level I trauma centres	Missing airway management data	274 to 335
Härtl (2006) [43]	Prospective cohort study	2000–2004	New York State, USA	Mechanism of injury consistent with TBI and GCS ≤ 8 for at least 6 h after injury	Death in ED or admitted with diagnosis of brain death, admission to study hospital >24h after injury, non-paralyzed patients with fixed and dilated pupils, missing pupil status, missing outcome assessment, GCS ≥ 9 on day 1, GCS motor score = 6 on any day, transport time < 10 minutes	1123^e^
Irvin (2010) [44]	Retrospective cohort study. Only patients with isolated head injury are considered	2000–2005	numerous locations throughout the USA and Puerto Rico	GCS = 3 and H-AIS score assigned	Received paralytics or sedatives in the field, missing data for several predefined variables	1504^f^
Karamanos (2014) [45]	Retrospective matched cohort study	2003–2011	Los Angeles County, USA	H-AIS ≥ 3 and/or GCS ≤ 8	Extra-cranial AIS ≥ 3, cardiac arrest in the field, lack of immediate ABG obtained at admission	220^c^
Klemen (2006) [46]	Cohort study	Intervention: 2000–2004; Controls: 1998–2004	Maribor, Slovenia	GCS ≤ 8, H-AIS > 3, ISS > 15	NR	124
Lenartova (2007) [47]	Prospective cohort study	1999–2004	five locations throughout Austria	GCS ≤ 8 following resuscitation or GCS score deteriorating to ≤ 8 within 48 hours of injury	Death on scene, death during transport to hospital or immediately after admission to the emergency room	393
Murray (2000) [48]	Retrospective cohort study, unmatched and matched analyses reported	1995–1997	Los Angeles County, USA	Field GCS ≤ 8 and H-AIS ≥ 3	Missing documentation for outcome and intubation status, unsuccessful intubation excluded in some of the sub-analyses	114 to 852
Poste (2004) [49]	Matched cohort study, prospectively enrolled intervention group matched to historical controls.	Intervention: 1998–2002; Controls: NR	San Diego County, USA	See Davis (2003) + ground transport or air medical transport depending on sub-analysis	Inability to obtain iv access, CPR before administration of RSI medication, H-AIS < 2 or higher H-AIS defined by neck injury, inability to intubate, primary airway management by air-medical crew	237 to 771^d^
Singbartl (1985) [50]	Prospective cohort study	NR	Bochum, Germany	Cerebral trauma, GCS ≤ 7	NR	147
Sloane (2000) [51]	Retrospective cohort study	Intervention: 1988–1995; Controls: 1992–1995	San Diego County, USA	Adult trauma patients, GCS ≤ 8, ISS ≥ 9, H-AIS ≥ 3, all other AIS ≤ 3	Incomplete records, non-RSI, nasotracheal intubation, cricothyrotomy, intubation before arrival of aeromedical crews, interhospital transfer	75^d^
Tuma (2014) [52]	Retrospective cohort study	2008–2011	Qatar	Age >14 years, field GCS ≤ 8 and H-AIS ≥ 3 and all other AIS ≤ 3	Death within 24 hours due to haemorrhage or unclear cause, patients transferred from other hospital, intubation in OR or ICU	160
Vandromme (2011) [53]	Cohort study	2006–2009	Birmingham, Alabama, USA	Blunt mechanism, GCS ≤ 8 and CT-confirmed TBI, defined as Marshall Score of II-V	NR	135
Wang (2004) [55]	Retrospective cohort study	2000–2002	Pennsylvania, USA	Age ≥ 18, trauma with ICD-9-CM injury classification 800–995, H-AIS ≥ 3	Interhospital transfer, no treatment by advanced life support rescuers, not intubated either in the field or in the ED	4098
Wang (2014) [54]	Secondary analysis of a prospective RCT on hypertonic fluid resuscitation.	2006–2009	multiple locations throughout the USA and Canada	Age ≥ 15 years, blunt mechanism of injury, GCS ≤ 8	Shock, pregnancy, out-of-hospital CPR, more than 2000 ml of crystalloid or any colloid or blood products prior to enrolment, severe hypothermia, drowning, asphyxia due to hanging, burns of more that 20% total body surface area, isolated penetrating head injury, inability to obtain venous access, prisoner status, intrafacility transfers, >4 h time interval between dispatch call and study intervention, death in the field or ED, neither advanced airway management in the field nor in the ED, missing key covariates	1116
Winchell (1997) [56]	Retrospective cohort study	1991–1995	San Diego County, USA	Blunt mechanism, GCS ≤ 8, admission to ICU or hospitalization for more than 3 days or death, depending on sub-analysis also H-AIS ≥ 4 and all other AIS ≤ 3, GCS = 3 or GCS 4–8. Intervention: apnoea or ineffective ventilation, no gag reflex.	Depending on sub-analysis: transport by ground or air	50 to 1092^d^

^a^ In- and exclusion criteria for the population of interest.

^b^ Number of population on which the analyses of interest (prehospital intubation versus no intubation in TBI patients) are based. If multiple analyses are presented in the manuscript, the range of the number of patients used in the analyses is reported.

^c^ Data from the studies by Bukur and Karamanos report patients from the same region and overlapping time period.

^d^ Data from the studies by Davis, Poste, Sloane and Winchell are all from the same region and overlapping time periods and partially report overlapping data.

^e^ Total number of patients in study, unclear whether all are included in analysis of interest.

^f^ Data are from the National Trauma Data Bank and might include some patients that have also been included to other studies that have been performed in the USA.

ABG: arterial blood gas

(H-)AIS: (head) abbreviated injury scale

CPR: cardiopulmonary resuscitation

CT: computed tomography

ED: emergency department

GCS: Glasgow Coma Scale

ICU: intensive care unit

ICD-9-CM: international classification of diseases, 9^th^ revision, clinical modification

ISS: injury severity scale

NR: not reported

OR: operating room

RCT: randomized controlled trial

RSI: rapid sequence induction

TBI: traumatic brain injury

One of the studies is an RCT, and one study is a secondary cohort-analysis of an RCT that had been performed to address a different research question. All other studies are cohort studies (Table 1).

The Cochrane Collaboration’s tool revealed a low risk of bias for the RCT. The Newcastle-Ottawa Scale rating ranged between four and nine stars for the cohort studies, with a median rating of 7 stars (Table 2).

**Table 2:** Quality Assessment.

First author (year)	Newcastle-Ottawa Quality Assessment Scale									Cochrane Collaboration’s tool for assessing risk of bias							Meta-Analysis	
	Selection			Comparability			Outcome			A	B	C	D	E	F	G	Eligible	Selected
	**1**	**2**	**3**	**4**	**5**	**6**	**7**	**8**	**9**									
Bernard (2010) [33]										low	low	NA	low	low	low	low	Yes	Yes
Bochicchio (2003) [34]	*	*	*	*	-	-	*	-	-								No	No
Bukur (2011) [35]	*	*	*	*	*	*	*	-	-								Yes	No^a^
Davis (2003) [37]	-	-	*	*	*	*	*	*	*								No	No
Davis (2004) [36]	-	-	*	*	*	*	*	-	*								No	No
Davis (2005a) [39]	*	-	*	*	*	*	*	-	*								No	No
Davis (2005b) [40]	*	*	*	*	*	*	*	-	*								Yes	Yes^b^
Davis (2005c) [41]	-	-	*	*	*	*	*	*	*								No	No
Davis (2006) [38]	*	*	*	*	-	-	*	-	*								No	No
Franschman (2011) [42]	*	*	*	*	*	*	*	-	*								Yes	Yes
Härtl (2006) [43]	*	*	*	*	*	*	*	*	-								No^c^	No
Irvin (2010) [44]	-	*	*	*	*	*	*	*	-								No^c^	No
Karamanos (2014) [45]	*	*	*	*	*	*	*	*	*								Yes	Yes
Klemen (2006) [46]	*	*	*	*	(*)^d^	(*)^d^	*	*	*								No	No
Lenartova (2007) [47]	*	*	*	*	-	-	*	*	*								No	No
Murray (2000) [48]	*	*	*	*	(*)^e^	(*)^e^	*	*	*								Yes	Yes^b^
Poste (2004) [49]	-	-	*	*	*	*	*	*	*								No	No
Singbartl (1985) [50]	*	-	*	*	-	-	*	-	*								No	No
Sloane (2000) [51]	-	-	*	*	-	-	*	*	-								No	No
Tuma (2014) [52]	*	*	*	*	*	*	*	*	*								Yes	Yes
Vandromme (2011) [53]	*	*	*	*	*	*	*	-	-								No^c^	No
Wang (2004) [55]	*	*	*	*	*	*	*	*	*								No^c^	No
Wang (2014) [54]	*	*	*	*	*	*	*	*	*								No^c^	No
Winchell (1997) [56]	*	*	*	*	-	-	*	*	*								No	No

^a^ Not selected because of potential overlap with Karamanos (2014).

^b^ Several analyses described in the manuscript were eligible; the one with the smallest standard error of the estimated OR was selected.

^c^ Study eligible based on quality criteria, but EMS-provider experience was “indeterminate” (see Table 3).

^d^ Several analysis presented; first hour survival and first day survival data are adjusted, however the analysis with the outcome of main interest (hospital mortality) is not adjusted.

^e^ Several analyses are presented, among which one matched and one adjusted analyses. Both of these analyses earned two stars for comparability, while the cohorts are not comparable in the crude analyses.

Newcastle-Ottawa Quality Assessment Scale

Selection:     1. Representativeness of the exposed cohort (prehospital intubation)

2. Selection of the non-exposed cohort (no prehospital intubation)

3. Ascertainment of exposure

4. Demonstration that outcome of interest was not present at start of study

Comparability:     5. Comparability of cohorts on the basis of the design or analysis: most important factor

6. Comparability of cohorts on the basis of the design or analysis: additional factors

Outcome:     7. Assessment of outcome

8. Was follow-up long enough for outcomes to occur?

9. Adequacy of follow up of cohorts

Cochrane Collaboration’s tool for assessing risk of bias: Domains

A. Sequence generation

B. Allocation concealment

C. Blinding of participants and personnel

D. Blinding of outcome assessors

E. Incomplete outcome data

F. Selective outcome reporting

G. Other sources of bias

### Patient and injury characteristics

Patients were predominantly male and typically had a mean or median age of around 35 to 45 years. In accordance with our study selection criteria, markers of injury severity generally reflect serious injury (Table 3).

**Table 3:** Patient and injury characteristics^a^.

First author (year)	Patient age^b^	Male gender (%)	Isolated TBI?	Initial GCS^b^	H-AIS^b^	ISS^b^
Bernard (2010) [33]	Intervention: 40.0 ± 22	Intervention: 75	No	Intervention: 5 (3–7)	Intervention: 4.0 ± 1.4	Intervention: 30.5 ± 14.8
	Control: 41.4 ± 23	Control: 77		Control: 5 (3–7)	Control: 3.9 ± 1.4	Control: 30.1 ± 14.5
Bochicchio (2003) [34]	Intervention: 35 ± 21	Overall: 81	No	Intervention: 4.0 ± 0.8	Intervention: 4.9 ± 0.7	Intervention: 20.1 ± 8
	Control: 40 ± 15			Control: 4.4 ± 2.1	Control: 4.5 ± 0.9	Control: 19.2 ± 9
Bukur (2011) [35]	Intervention: 35.9 ± 18.2	Intervention: 82	Yes	Intervention: 3.3 ± 1.1	Intervention: 4.8 ± 0.5	Intervention: 26.7 ± 8.4
	Control: 38.1 ± 24.2	Control: 76		Control: 11.7 ± 4.2	Control: 4.0 ± 0.8	Control: 18.4 ± 7.0
Davis (2003) [37]	Intervention: 37.1	Intervention: 81	No	NR	Intervention: 3.91	Intervention: 27.6
	Control: 36.8	Control: 81			Control: 3.92	Control: 26.3
Davis (2004) [36]	Intervention: 38.1	Intervention: 81	No	NR	Intervention: 3.92	Intervention:26.2
	Control: 36.9	Control: 81			Control: 3.92	Control: 26.6
Davis (2005a) [39]	Intervention. 33.0	Intervention: 79	No	Intervention: 4.1	Intervention: 4.42	Intervention: 32.9
	Control: 37.5	Control: 78		Control: 4.6	Control: 4.42	Control: 31.2
Davis (2005b) [40]	Intervention: 35.3	Overall: 76	No	Intervention: 4.4	Intervention: 4.6	Intervention: 36.6
	Control: 37.6^c^			Control: 8.0^c^	Control: 4.2^c^	Control: 28.3^c^
Davis (2005c) [41]	Intervention: 37.1	Intervention: 81	No	NR	Intervention: 3.91	Intervention: 26.7
	Control: 37.8	Control: 81			Control: 3.91	Control 27.5
Davis (2006) [38]	Intervention: 35.4	Intervention: 79	No	Intervention: 4.5	Intervention: 4.5	Intervention: 34.0
	Control: 40.2	Control: 77		Control: 10.3	Control: 3.9	Control: 24.4
Franschman (2011) [42]	Intervention: 43 ± 21	Overall: 70	No	Intervention: 3 (3–3)	NR	Intervention: 32 (25–41)
	Control: 48 ± 20			Control: 5 (3–7)		Control: 25 (22–29)
Härtl (2006) [43]	Overall: 36.0 ± 20.6	Overall: 75	No	Overall:	NR	NR
				GCS 3–5: 53.7%		
				GCS 6–8: 33.3%		
				GCS ≥ 9: 13.0%		
Irvin (2010) [44]	Intervention: 37.9 ± 20.8	NR	Yes	NR (should be 3, see inclusion criteria)	NR	Intervention: 31.6 ± 16.2
	Control: 37.7 ± 20.0					Control: 24.2 ± 16.0
Karamanos (2014) [45]	Intervention: 35.3 ± 1.3	Intervention: 86	Yes	NR	NR	Intervention:
						ISS ≤ 15: 5.5%
						ISS = 16–24: 18.2%
						ISS ≥ 25: 76.4%
	Control: 36.2 ± 1.5	Control: 89				Control:
						ISS ≤ 15: 8.5%
						ISS = 16–24: 18.8%
						ISS ≥ 25: 72.7%
Klemen (2006) [46]	Intervention: 44.8 ± 23.6	Intervention: 77	No	Intervention: 5 (3–8)	NR	Intervention: 24 (16–26)
	Control: 42.5 ± 21.3	Control: 82		Control: 6 (4–8)		Control: 23 (17–25)
Lenartova (2007) [47]	Overall: 48.9 ± 20.8	Overall: 72	No	Overall: 5.6 ± 2.9	NR	Overall: 27.0 ± 12.7
Murray (2000) [48]	Intervention: 34	Intervention: 70	No	NR	Intervention:	Intervention: 29.6
					H-AIS = 3: 15%	
					H-AIS = 4: 15%	
					H-AIS = 5: 65%	
					H-AIS = 6: 5%	
	Control: 34	Control: 78			Control:	Control: 26.7
					H-AIS = 3: 17%	
					H-AIS = 4: 30%	
					H-AIS = 5: 52%	
					H-AIS = 6: 1%	
	Attempted Intubation: 33	Attempted Intubation: 79			Attempted Intubation:	Attempted Intubation: 31.8
					H-AIS = 3: 2%	
					H-AIS = 4: 23%	
					H-AIS = 5: 72%	
					H-AIS = 6: 4%	
Poste (2004) [49]	*Air transport cohort*	*Air transport cohort*	No	*Air transport cohort*	*Air transport cohort*	*Air transport cohort*
	Intervention: 38.0	Intervention: 79		Intervention: 4.9	Intervention: 3.91	Intervention: 27.2
	Control: 38.4	Control: 79		Control: NR	Control: 3.92	Control: 28.0
	*Ground transport cohort*	*Ground transport cohort*		*Ground transport cohort*	*Ground transport cohort*	*Ground transport cohort*
	Intervention: 37.2	Intervention: 81		Intervention: 4.9	Intervention: 3.91	Intervention: 26.4
	Control: 37.8	Control: 81		Control: NR	Control: 3.91	Control: 27.1
Singbartl (1985) [50]	Overall: 41.2	NR	No	Intervention:	NR	NR
				GCS 3: 23.7%		
				GCS 4–5: 55.9%		
				GCS 6–7: 20.4%		
				Control:		
				GCS 3: 20.4%		
				GCS 4–5: 46.3%		
				GCS 6–7: 33.3%		
Sloane (2000) [51]	Intervention: 26.2	Intervention: 76	Yes	Intervention: 5.2	Intervention: 4.8	Intervention: 31.4
	Control: 36.2	Control: 81		Control: 5.8	Control: 4.7	Control: 29.0
Tuma (2014) [52]	Intervention: 30 ± 14	Intervention: 95	Yes	Intervention: median Glasgow motor score = 1	NR	Intervention: 28 ± 8
	Control: 34 ± 15	Control: 98		Control: median Glasgow motor score = 3		Control: 27 ± 10
Vandromme (2011) [53]	Overall: 38.0	Overall: 77	No	Intervention: 4.1	Intervention: 4.4	Intervention: 38.0
				Control: 5.9	Control: 4.6	Control: 33.7
Wang (2004) [55]	Intervention:	Intervention: 74	No	NR	Intervention:	Intervention:
	18–30 years: 41.2%				H-AIS = 3: 18.5%	ISS < 10: 1.1%
	31–40 years: 17.1%				H-AIS = 4: 25.1%	ISS = 10–15: 5.3%
	41–50 years: 15.1%				H-AIS = 5: 53.6%	ISS = 16–25: 23.3%
	51–60 years: 8.4%				H-AIS = 6: 2.7%	ISS = 26–35: 36.9%
	61–70 years: 6.8%					ISS = 36–50: 25.3%
	71–80 years: 6.3%					ISS = 51–70: 4.3%
	>80 years: 4.8%					ISS > 70: 3.7%
	Control:	Control: 75			Control:	Control:
	18–30 years: 33.6%				H-AIS = 3: 28.0%	ISS < 10: 3.9%
	31–40 years: 15.2%				H-AIS = 4: 31.1%	ISS = 10–15: 9.9%
	41–50 years: 16.4%				H-AIS = 5: 39.7%	ISS = 16–25: 35.1%
	51–60 years: 9.5%				H-AIS = 6: 1.2%	ISS = 26–35: 34.2%
	61–70 years: 7.8%					ISS = 36–50: 13.4%
	71–80 years: 9.8%					ISS = 51–70: 1.8%
	>80 years: 7.3%					ISS > 70: 1.7%
Wang (2014) [54]	Intervention: 38.3 ± 18.1	Intervention: 77	No	Intervention: 5.0 ± 2.4	Intervention: 3.8 ± 1.5	Intervention: 29.4 ± 15.4
	Control: 40.1 ± 19.0	Control: 77		Control: 5.5 ± 2.4	Control: 3.4 ± 1.9	Control: 24.9 ± 14.8
Winchell (1997) [56]	Intervention: 32.6	NR	No/Yes^d^	Intervention: 4.8	Intervention: 3.9	Intervention: 27
	Control: 33.5			Control: 4.6	Control: 3.6	Control: 25

^a^ For studies presenting data from several patient populations or several sub-analyses, the reported patient characteristics refer to the total patient population.

^b^ Presented as mean, mean ± SD, mean (95% CI), median, median (IQR) or as percentage per category, as reported by the authors or as calculated from the available data.

^c^ Multiple analyses with two different control groups (no prehospital invasive airway management, intubation in the emergency department) performed in the study. The presented data are for the subpopulation of patients intubated in the emergency department.

^d^ Study reports sub-analyses for patients with isolated TBI.

GCS: Glasgow Coma Scale; H-AIS: head abbreviated injury scale; ISS: injury severity scale; NR: not reported; TBI: traumatic brain injury

### Treatments and level of experience of EMS providers

While all identified studies basically compared PHI versus no PHI, there were differences in how the intervention and control groups were defined. A portion of the analyses included intubation attempts or use of alternative airway devices after failed intubation in an intention-to-treat approach, while such attempts were excluded or not specifically reported in other analyses. In the PHI groups, patients were sometimes intubated with a “rapid sequence induction” approach using anaesthetic drugs and neuromuscular blocking agents, while patients in other studies were intubated without such medication, or medication use was not reported. The control group generally consisted of patients who were not intubated in the prehospital setting. However, a part of the studies specifically defined the control group as patients who required endotracheal intubation in the hospital. Table 4 summarizes treatments in the intervention- and control groups per study.

**Table 4:** Treatments.

First author (year)	Intervention	Control	Medication used for prehospital intubation	Intubated by	Level of training
Bernard (2010) [33]	Prehospital RSI (including attempts in an intention-to-treat approach)	Hospital intubation (prehospital intubation permitted if airway reflexes lost during transport)	Fentanyl, midazolam, succinylcholine, atropine if heart rate < 60/min, minimum 500 ml lactated Ringer’s solution. After intubation: pancuronium, morphine infusion, midazolam infusion	Specially trained intensive care paramedics	Extended
Bochicchio (2003) [34]	Prehospital RSI	ED intubation	Midazolam (may be omitted), lidocaine, succinylcholine. After intubation: vecuronium if significant resistance to ventilation occurs	Ground paramedics and flight paramedics	Indeterminate
Bukur (2011) [35]	Prehospital intubation	ED intubation	NR	Paramedics	Limited
Davis (2003) [37]	Prehospital RSI or cricothyrotomy (after 3 unsuccessful intubation attempts)	No prehospital intubation	Midazolam if SBP > 120 mmHg, succinylcholine. After intubation: rocuronium, additional midazolam after 30 min if SBP remained > 120 mmHg, morphine every 10 minutes if SPB > 140 mmHg and heart rate > 100 BPM	Paramedics. A portion of intubations may have been performed by flight nurses or emergency medicine resident physicians	Indeterminate
Davis (2004) [36]	Prehospital RSI, combitube as salvage device or cricothyrotomy (after 3 unsuccessful intubation attempts)	No prehospital intubation	Midazolam if SBP > 120 mmHg, succinylcholine. After intubation: rocuronium, morphine every 10 minutes if SPB > 140 mmHg and heart rate > 100 BPM	Paramedics	Limited
Davis (2005a) [39]	Prehospital intubation by aeromedical teams, patients transported by helicopter	ED intubation, patients transported by ground ambulance	Patients may have been intubated using RSI medication; no details provided	Flight crews (certified flight nurses and emergency medicine resident physicians)	Extended
Davis (2005b) [40]	Prehospital tracheal intubation (depending on sub-analysis in- or excluding combitube, cricothyrotomy or nasotracheal intubation)	No prehospital invasive airway management	A portion of the patients (especially those intubated by flight crews) may have been intubated using RSI medication; no details provided	Depending on sub-analysis: Paramedics or paramedics and flight crews (flight nurses and emergency medicine resident physicians)	Indeterminate or limited depending on the sub-analysis
Davis (2005c) [41]	Prehospital RSI	No prehospital intubation	Midazolam if SBP > 120 mmHg, succinylcholine. After intubation: rocuronium additional midazolam after 30 min if SBP remained > 120 mmHg, morphine every 10 minutes if SPB > 140 mmHg and heart rate > 100 BPM	Paramedics, a portion of intubations were performed by flight nurses or emergency medicine residents	Indeterminate
Davis (2006) [38]	Prehospital invasive airway management including intubation, combitube insertion or cricothyrotomy	No prehospital invasive airway management	A portion of the patients were intubated using NMBA	Paramedics, a portion of intubations were performed by flight nurses or emergency medicine residents	Indeterminate
Franschman (2011) [42]	Prehospital intubation	No prehospital intubation	Different regimes, with or without RSI medications	Emergency physicians or ambulance nurses	Extended
Härtl (2006) [43]	Prehospital intubation	No prehospital intubation	NR	NR	Indeterminate
Irvin (2010) [44]	Prehospital intubation	No prehospital intubation	No sedatives or paralytic agents	NR	Indeterminate
Karamanos (2014) [45]	Prehospital intubation	No prehospital intubation, oxygen by mask	NR	Paramedics	Limited
Klemen (2006) [46]	Prehospital RSI	No prehospital RSI	Various anaesthetic induction agents with or without succinylcholine	Emergency physicians	Extended
Lenartova (2007) [47]	Prehospital intubation	No prehospital intubation	NR	Predominantly emergency physician led teams (96%)	Extended
Murray (2000) [48]	Prehospital intubation (unsuccessful attempts either in- or excluded depending on sub-analysis)	No prehospital intubation (unsuccessful attempts either in- or excluded depending on sub-analysis)	None	Paramedics	Limited
Poste (2004) [49]	Successful prehospital RSI with endotracheal tube or combitube (after a maximum of 3 unsuccessful intubation attempts)	No prehospital intubation	Midazolam if SBP > 120 mmHg, succinylcholine. After intubation: rocuronium, morphine every 10 minutes if SPB > 140 mmHg and heart rate > 100 BPM	Paramedics	Limited
Singbartl (1985) [50]	Prehospital intubation	No prehospital intubation	NR	Emergency physicians and paramedics	Indeterminate
Sloane (2000) [51]	Prehospital RSI by aeromedical crews	ED RSI, transport by ground ambulance	Lidocaine, consider fentanyl, succinylcholine. After intubation consider vecuronium and fentanyl	Aeromedical physicians or flight nurses	Extended
Tuma (2014) [52]	Prehospital intubation	ED intubation	RSI, no details reported	Well trained critical care paramedics	Extended
Vandromme (2011) [53]	Prehospital intubation	ED intubation	NR	NR	Indeterminate
Wang (2004) [55]	Prehospital intubation, including combitube, cricothyrotomy or tracheotomy	ED intubation	Different regimes, with or without NMBA	Paramedics, flight paramedics, nurses, physicians	Indeterminate
Wang (2014) [54]	Prehospital advanced airway management including intubation, insertion of supraglottic airway devices or surgical airways	ED advanced airway management	Different regimes, with or without NMBA	NR	Indeterminate
Winchell (1997) [56]	Prehospital intubation. Ground paramedics: max. 3 attempts, aeromedical teams: cricothyrotomy if intubation could not be performed	No prehospital intubation	Either none (ground paramedics) or NMBA (flight crews)	Either paramedics or aeromedical crews (flight nurses, flight paramedics, occasionally physicians)	Indeterminate

BPM: beats per minute

ED: emergency department

NMBA: neuromuscular blocking agents

NR: not reported

RSI: rapid sequence induction

SBP: systolic blood pressure

In seven studies, EMS-providers with extended experience performed PHI, and EMS-provider experience was considered limited in five studies (Table 4). Twelve studies were scored as “indeterminate” experience. One of these studies reported sub-analyses for a subset of patients intubated by EMS-personnel with limited experience.

### Summary of results from individual studies

In the 24 studies included in the systematic review, the observed unadjusted OR point estimates ranged between 0.12 and 64.7, while the adjusted estimates ranged between 0.38 and 5.0. In studies where PHI was performed by providers with limited level of training, reported adjusted ORs were between 1.96 and 5.0. In contrast, adjusted ORs were in the range between 0.38 and 0.87 when experienced providers performed intubation. Table 5 reports mortality data of individual studies and lists factors that have been used to adjust the estimates in the different studies.

**Table 5:** Mortality.

First author (year)	Time of mortality assessment	Sub-Analysis	Intervention		Control		Unadjusted OR (95% CI)	Adjusted OR (95% CI)^d^	Factors used for adjustment or matching
			Alive	Dead	Alive	Dead			
Bernard (2010) [33]	Hospital discharge		107	53	97	55	0.87 (0.55–1.39)	Considered equivalent to unadjusted	(Randomized Controlled Trial)
Bochicchio (2003) [34]	NR^a^		60^c^	18^c^	99^c^	14^c^	2.12 (0.98–4.58)	NR	NA
Bukur (2011) [35]	NR^a^		6^c^	55^c^	2019^c^	286^c^	64.7 (27.6–151.6)	5.0 (1.7–13.7)	Mechanism of injury, admission SBP, admission GCS, H-AIS, ISS
Davis (2003) [37]	Hospital discharge		140^c^	69^c^	475^c^	152^c^	1.54 (1.09–2.17)	1.6 (NR)	Adjustment: age, sex, H-AIS, Chest-AIS, Abdomen-AIS, scene time, admission SBP. Matching: age, sex, mechanism of injury, trauma centre, ISS, H-AIS, face AIS, chest AIS, abdomen AIS, extremities AIS, skin AIS
Davis (2004) [36]	Hospital discharge		35^c^	24^c^	139^c^	38^c^	2.51 (1.33–4.72)	NR	Age, sex, mechanism of injury, trauma centre, ISS, H-AIS, face AIS, chest AIS, abdomen AIS, extremities AIS, skin AIS
Davis (2005a) [39]	NR^a^		719	531	565	428	0.97 (0.82–1.15)	0.70 (0.56–0.88)	Age, sex, mechanism of injury, preadmission hypotension, H-AIS, ISS, pre-intubation GCS
Davis (2005b) [40]	NR^a^	Population H-AIS ≥ 3. Control: no invasive PH airway management	974	1256	6053	1220	6.40 (5.77–7.10)	2.78 (2.38–3.13)	Age, sex, mechanism of injury, preadmission hypotension, H-AIS, ISS, pre-intubation GCS
		Population GCS ≤ 8 and H-AIS ≥ 3. Control: no invasive PH airway management	830	1221	1468	901	2.40 (2.12–2.71)	1.35 (1.15–1.59)	"
	NR^a^	Population H-AIS ≥ 4. Control: no invasive PH airway management	737	1163	3139	1103	4.49 (4.00–5.04)	1.39 (1.19–1.64)	"
		Population GCS ≤ 8 and H-AIS ≥ 4. Control: no invasive PH airway management	652	1132	1083	843	2.23 (1.95–2.54)	1.28 (1.09–1.52)	"
		Population H-AIS ≥ 3, excludes intubation by aeromedical crews. Control: no invasive PH airway management	250	695	4589	1063	12 (10.24–14.07)	2.38 (1.92–3.03)	"
		Population GCS ≤ 8 and H-AIS ≥ 3, excludes intubation by aeromedical crews. Control: no invasive PH airway management	229	687	1236	798	4.65 (3.90–5.53)	2.13 (1.69–2.63)	"
		Population H-AIS ≥ 4, excludes intubation by aeromedical crews. Control: no invasive PH airway management	196	641	2430	973	8.17 (6.85–9.74)	2.27 (1.82–2.86)	"
		Population GCS ≤ 8 and H-AIS ≥ 4, excludes intubation by aeromedical crews. Control: no invasive PH airway management	182	633	910	749	4.23 (3.49–5.12)	1.96 (1.56–2.5)	"
		Population H-AIS ≥ 3, Control: ED intubation	1024	1390	1296	537	3.28 (2.88–3.73)	2.13 (1.82–2.5)	"
	NR^a^	Population GCS ≤ 8 and H-AIS ≥ 3. Control: ED intubation	870	1351	646	396	2.53 (2.18–2.95)	1.47 (1.2–1.79)	"
		Population H-AIS ≥ 4. Control: ED intubation	769	1284	886	489	3.03 (2.62–3.49)	1.45 (1.2–1.75)	"
		Population GCS ≤ 8 and H-AIS ≥ 4. Control: ED intubation	679	1250	512	372	2.53 (2.15–2.98)	1.43 (1.16–1.75)	"
Davis (2005c) [41]	Hospital discharge		240^c^	112^c^	537^c^	167^c^	1.50 (1.13–1.99)	2.0 (1.4–2.8)	Adjustment: age, sex, arrival SBP, H-AIS, ISS. Matching: age, sex, mechanism of injury, trauma centre, ISS, H-AIS, AIS for face, chest, abdomen, extremities and skin
Davis (2006) [38]	NR^a^		447	443	2368	546	4.30 (3.66–5.05)	NR	NA
Franschman (2011) [42]	Hospital discharge^b^		132	101	60	42	1.09 (0.68–1.75)	0.63 (0.27–1.49)	Age, ISS, GCS, pupillary reflex, hypoxia, hypotension
Härtl (2006) [43]	Two week mortality		NR	NR	NR	NR	NR	0.82 (0.59–1.14)	Hypotension status on day 1, age category, pupil status on day 1, GCS (unclear whether initial value or at day 1)
Irvin (2010) [44]	Hospital discharge		NR	NR	NR	NR	NR	1.99 (1.35–2.93)	ISS, age, penetrating trauma, improvement en route
Karamanos (2014) [45]	Hospital discharge ^b^		17	38	74	91	1.82 (0.95–3.48)	NR	Propensity scores calculated by logistic regression. All demographic and clinical variables that differed significantly between the groups were used in the model
Klemen (2006) [46]	Hospital discharge		39	25	35	25	0.90 (0.44–1.84)	NR	NA
	First day mortality		57	7	45	15	0.37 (0.14–0.98)	0.38 (0.26–0.55)	Age, gender, mechanism of injury, GCS, ISS, initial SaO_2_, SBP
	First hour mortality		62	2	47	13	0.12 (0.03–0.54)	0.45 (0.34–0.56)	"
Lenartova (2007) [47]	90 days after trauma		196^c^	128^c^	51^c^	18^c^	1.85 (1.03–3.31)	NR	NA
	ICU mortality		214^c^	110^c^	54^c^	15^c^	1.85 (1.00–3.43)	NR	NA
Murray (2000) [48]	Hospital discharge	Successful PH intubation vs. no prehospital intubation (as treated)	15	66	415	356	5.13 (2.88–9.14)	NR	NA
	"	PH intubation attempts vs. no prehospital intubation attempts (intention-to- treat)	25	113	405	309	5.92 (3.75–9.36)	4.18 (2.06–8.93)	Gender, GCS, H-AIS, ISS, transport mode, associated injuries, mechanism of injury
	"	Unsuccessful attempts excluded in both cohorts	15	66	405	309	5.77 (3.23–10.3)	NR	NA
	"	Matched cohorts, unsuccessful attempts excluded in both cohorts	9	48	17	40	2.27 (0.91–5.63)	NR	GCS, H-AIS, ISS group, significant associated injuries, age group, mechanism of injury, transport
Poste (2004) [49]	Hospital discharge	Ground transported patients	172^c^	85^c^	406^c^	108^c^	1.86 (1.33–2.60)	NR	Age, gender, mechanism of injury, trauma centre, ISS, H-AIS, AIS for face, chest, abdomen, extremities and skin
	"	Air transported patients	57^c^	22^c^	109^c^	49^c^	0.86 (0.47–1.56)	NR	"
Singbartl (1985) [50]	NR	NA	48	45	31	23	1.26 (0.64–2.48)	NR	NA
Sloane (2000) [51]	Within 30 days after trauma	NA	18	3	42	12	0.58 (0.15–2.32)	NR	NA
Tuma (2014) [52]	Within 30 days after admission	NA	48^c^	57^c^	38^c^	17^c^	2.65 (1.33–5.29)	0.55 (0.24–1.26)	Age, ISS, motor GCS, EMS time
Vandromme (2011) [53]	NR	NA	34^c^	30^c^	42^c^	29^c^	1.28 (0.65–2.53)	NR	ED-GCS, ED SBP, ISS
Wang (2004) [55]	Hospital discharge	NA	926	871	1652	649	2.39 (2.10–2.73)	3.99 (3.21–4.93)	Age, sex, H-AIS, ISS, other severe injury, admission SBP, mechanism of injury, use of neuromuscular blocking agents, and a propensity score summarizing selected pre-existing medical conditions, social variables and in-hospital events.
Wang (2014) [54]	Within 28 days of after trauma	NA	558	206	259	93	1.03 (0.77–1.37)	1.57 (0.93–2.64)	Age, sex, ISS, mechanism of injury, initial SBP, initial GCS, highest field heart rate, out-of-hospital neuromuscular blockade use, mode of transport, H-AIS, parent trial intervention arm, study site
Winchell (1997) [56]	Hospital discharge	GCS ≤ 8, ground transport	418	147	336	191	0.62 (0.48–0.80)	NR	NA
	"	GCS ≤ 8 and H-AIS ≥ 4, ground transport	249	138	121	163	0.41 (0.30–0.56)	"	"
	"	GCS ≤ 8 and H-AIS ≥ 4, isolated TBI, ground transport	159	47	73	72	0.30 (0.19–0.47)	"	"
	"	GCS = 3 and H-AIS ≥ 4, isolated TBI, ground transport	53	37	27	59	0.32 (0.17–0.59)	"	"
	"	GCS = 4–8 and H-AIS ≥ 4, isolated TBI, ground transport	106	10	46	13	0.33 (0.14–0.82)	"	"
	"	GCS ≤ 8, air transport	280^c^	151^c^	56^c^	15^c^	2.01 (1.10–3.68)	"	"
	"	GCS ≤ 8 and H-AIS ≥ 4, air transport	177^c^	134^c^	21^c^	8^c^	1.99 (0.85–4.62)	NR	NA
	"	GCS ≤ 8 and H-AIS ≥ 4, air transport	80^c^	36^c^	13^c^	4^c^	1.46 (0.45–4.80)	"	"
	"	GCS = 3 and H-AIS ≥ 4, isolated TBI, air transport	22^c^	24^c^	2^c^	2^c^	1.09 (0.14–8.42)	"	"
	"	GCS = 4–8 and H-AIS ≥ 4, isolated TBI, air transport	58^c^	12^c^	11^c^	2^c^	1.14 (0.22–5.81)	"	"

^a^ Time of mortality assessment is not explicitly mentioned. However, we strongly assume that it is hospital mortality because no follow-up beyond hospital discharge is reported. For the studies by Davis and colleagues, this assumption is further underlined by the fact that other studies that have been performed by the same study group in the same patient population also regularly report hospital mortality. Requests to the authors to clarify this issue have remained unanswered.

^b^ Personal communication by the first author.

^c^ Calculated from reported percentages. May not necessarily be exactly the actual number due to rounding or unreported omission of patients from the analysis.

^d^ Calculation of the adjusted odds ratio may not necessarily be based on the same number of patients used for calculation of the unadjusted OR (e.g., due to missing covariates in some patients).

(H-)AIS: (head) abbreviated injury scale

CI: confidence interval

ED: emergency department

EMS: emergency medical services

GCS: Glasgow Coma Scale

ICU: intensive care unit

ISS: injury severity scale

NA: not applicable

NR: not reported

OR: odds ratio

SBP: systolic blood pressure

TBI: traumatic brain injury

### Pooled results: Meta-analysis, meta-regression and sensitivity analyses

**Fig 2:**
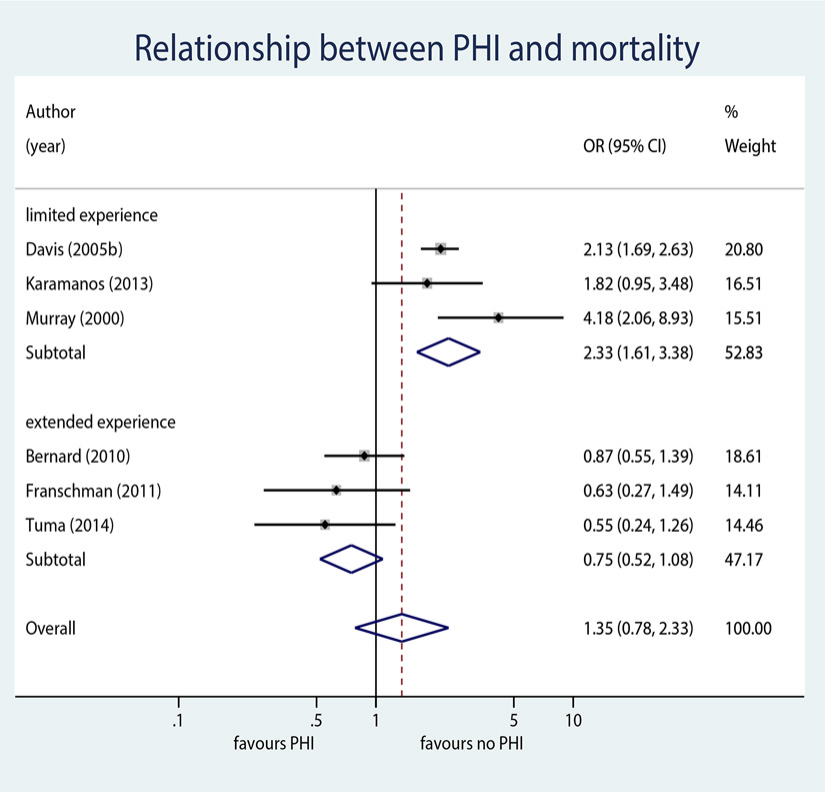
Forrest plot. Forrest plot summarizing the individual studies and pooled results of the meta-analysis. The relationship between prehospital intubation (PHI) and mortality is stratified by experience of prehospital healthcare providers.

Six analyses including data from 4772 patients met inclusion-criteria for the meta-analysis (Table 2). Overall, no significant association was observed between PHI and mortality (OR 1.35, 95% CI 0.78 to 2.33, p = 0.279, Fig 2). In studies in which intubation was performed by providers with limited experience, PHI was associated with higher odds of mortality (OR 2.33, 95% CI 1.61 to 3.38, p<0.001). In contrast, pooled results in the “extended experience” stratum showed no evidence for higher mortality in patients who were intubated in the prehospital setting (OR 0.75, 95% CI 0.52 to 1.08, p = 0.126). Meta-regression confirmed that EMS-provider experience is a significant predictor of mortality (p = 0.009). The funnel plot asymmetry regression test provided no evidence for small study bias (p = 0.312).

Substantial heterogeneity was observed between all studies (I^2^ = 83.3%). After adjusting for experience in the meta-regression, residual heterogeneity was negligible (I^2^ = 10.3%), suggesting that a large portion of the observed heterogeneity can be explained by differences in the level of experience.

We performed a sensitivity analysis excluding each of the studies one at a time and re-running the analyses without the excluded study. Odds-ratio estimates were of similar magnitude at each exclusion and all conclusions regarding significance remained the same, indicating that none of the included studies has undue influence on the overall results and conclusions (Table 6).

**Table 6:** Sensitivity analysis.

Study excluded	N total	Limited experience stratum		Extended experience stratum		Total		Heterogeneity I^2^		Metaregression
		OR (95% CI)	p-value	OR (95% CI)	p-value	OR (95% CI)	p-value	overall	residual	p-value
None	4772	2.33 (1.61–3.38)	< 0.001	0.75 (0.52–1.08)	0.126	1.35 (0.78–2.33)	0.279	83.3	10.3	0.009
Davis (2005b) [40]	1822	2.71 (1.20–6.12)	0.017	0.75 (0.52–1.08)	0.126	1.20 (0.60–2.37)	0.606	80.1	22.7	0.040
Karamanos (2013) [45]	4552	2.72 (1.44–5.12)	0.002	0.75 (0.52–1.08)	0.126	1.27 (0.65–2.45)	0.484	86.6	26.5	0.030
Murray (2000) [48]	3977	2.10 (1.70–2.58)	< 0.001	0.75 (0.52–1.08)	0.126	1.10 (0.62–1.97)	0.741	83.3	0.0	0.018
Bernard (2010) [33]	4460	2.33 (1.61–3.38)	< 0.001	0.59 (0.33–1.06)	0.079	1.49 (0.81–2.74)	0.195	80.5	11.9	0.030
Franschman (2011) [42]	4437	2.33 (1.61–3.38)	< 0.001	0.78 (0.52–1.17)	0.234	1.53 (0.87–2.71)	0.140	83.6	29.6	0.036
Tuma (2014) [52]	4612	2.33 (1.61–3.38)	< 0.001	0.81 (0.54–1.22)	0.314	1.58 (0.91–2.73)	0.103	82.1	20.8	0.031

CI: Confidence interval

OR: Odds ratio

## Discussion

### Summary of evidence

We performed a systematic review and meta-analysis to address effects of PHI on mortality in patients with severe TBI. The main finding is that effects of PHI depend on the level of experience of the EMS-providers who perform the intervention, and that PHI by EMS-personnel with limited experience in performing PHI is associated with increased mortality. When intubation is performed by well-trained personnel, we noted a trend towards improved survival, but the current evidence is insufficient to conclude that PHI by highly trained personnel reduces mortality.

### Strengths and limitations

Strengths of this systematic review include the comprehensive search strategy in three major databases without any restrictions, resulting in selection of 24 studies reporting data from more than 30,000 patients. The review was performed using pre-specified procedures according to published recommendations [24–26], and extracted data were double-checked to ensure accuracy of the reported information. A sensitivity analysis was used to confirm that the results and conclusions reflect the current overall literature rather than being the result of undue influence of any individual study. Meta-regression was used as an adjunct to the stratified meta-analysis to formally determine the significance of EMS-provider experience.

The included studies were—except for one RCT—mostly cohort studies. Such studies are subject to inherent limitations of observational research, but generally provide similar estimates of treatment effects as RCTs if they are well designed [57, 58]. We included all observational studies in the systematic review to give a comprehensive overview of previously published literature, but we only pooled data across studies that met pre-specified quality criteria. This approach allowed to quantitatively summarize the best available evidence while precluding bias due to limited data quality.

We focused on mortality as outcome because it is of high clinical relevance and unambiguously defined. Other outcomes such as incidence of complications or functional neurologic recovery in survivors are also relevant, but are not consistently reported. The manuscripts that do report complications report different kinds of complications, precluding meaningful comparisons across studies. Functional recovery was infrequently reported and different ordinal scales or dichotomous scores such as “good” or “favourable” outcome were used. Follow-up periods for assessment of functional recovery were extremely variable, ranging from hospital discharge [37, 38, 41, 55, 56] to one year after the trauma [47], which additionally complicates comparison of functional outcome data across studies.

In this context, it must be mentioned that timing of mortality assessment was also not the same across studies. Among the six studies included in our meta-analysis, five report hospital mortality [33, 40, 42, 45, 48] and one reports mortality within 30 days [52]. We believe that these data can be meaningfully combined, because most patients who die in hospital commonly die within 30 days, and most patients who survive until hospital discharge will likely be alive at 30 days. As the sensitivity analysis shows, excluding the study that uses 30-day mortality and using only those studies that report hospital mortality would not alter any of the conclusions on the relationship between PHI and mortality.

A limitation of our systematic review is that the patient populations and treatments were not exactly the same across studies. We included not only studies reporting patients with confirmed TBI, but also studies in which TBI was suspected based on the GCS score in combination with trauma mechanism and/or and clinical findings. Prehospital healthcare providers do not know the actual diagnosis but have to treat patients based on the suspected diagnosis, and therefore, including these studies makes the results more applicable to the real-life situation. Other differences in in- and exclusion criteria, geographical differences, and differences in how the intubations were performed (with or without anaesthetic drugs) can all introduce heterogeneity and bias. We therefore used a random-effects model for the meta-analysis to accommodate for such heterogeneity, and indeed, substantial heterogeneity was observed. However, most of the heterogeneity vanished after adjusting for EMS-provider experience in the meta-regression, indicating that experience is the single most important factor in explaining those differences between the studies that are not attributable to chance.

It was necessary to assign a level of experience to each study. Experience is rather abstract and difficult to quantify, and we therefore used the pragmatic approach to dichotomize experience as “limited” or “extended”. To avoid bias due to misclassification, three investigators performed this assessment and a level was assigned by unanimous consensus. With this careful approach, we can exclude that any study in which EMS-provider experience was actually “limited” may have been misclassified as “extended” experience or vice versa. The sensitivity analysis suggests that the conclusions of our study are robust against a possible misclassification of any study that should in fact actually have been classified as “indeterminate”.

The funnel plot regression asymmetry test did not provide evidence for small study bias. However, due to the rather small number of studies included in the meta-analysis and the limited power of such tests to detect bias at this sample size, we cannot completely exclude the possibility that our results might be affected by small study bias.

### Clinical implications

According to the “ABCDE” (Airway, Breathing, Circulation, Disability, Exposure) approach used in advanced trauma life support, securing the airway is a top priority in trauma patients with a threatened airway [59]. Patients with severe TBI have a high incidence of airway obstruction and hypoxia at the accident scene [10, 11], and there is a broad consensus that adequate prehospital airway management is crucial to prevent secondary injury [8]. Traditionally, early endotracheal intubation–as the gold standard of airway management–has been advocated for TBI patients with a GCS score ≤ 8, but this dogma is currently being challenged by publications that suggest higher mortality in patients who are prehospitally intubated. Von Elm and colleagues have previously addressed the relationship between PHI and mortality in a systematic review that included studies published up to 2007, and found that the available evidence was insufficient to allow recommendations on whether patients should or should not be intubated in the field [22]. Since then, several studies on the topic have been published. Our systematic review contains eight studies published after 2007, and four of the six studies in our meta-analysis were published in the last five years. Moreover, the previous systematic review did not address possible effects of EMS-provider experience, warranting the present investigation.

In line with the conclusions by Von Elm and colleagues, the pooled overall result of our meta-analysis provides no evidence for or against PHI, and does not allow an answer to the general question whether patients with severe TBI should be intubated in the field. However, we believe that the general overall effect is clinically less relevant because PHI may likely be either beneficial or detrimental, depending on the way it is performed, depending on side effects and complications, and depending on the ventilation strategy following intubation. Hence, an answer to the question whether or not to perform PHI should address additional factors that have previously received insufficient attention.

We hypothesized that the intubation skill of the EMS-provider is such a factor, because the incidence of adverse events related to poor intubation and ventilation performance may likely be higher when the intervention is performed by providers who are not well trained to do so. Indeed, our data provide strong evidence that PHI by EMS-providers with limited experience in performing intubations is associated with an approximately twofold increase in the odds of mortality. This suggests that the practice of routinely intubating patients with severe TBI should be abandoned in EMS systems in which providers have limited skills in performing this intervention.

We did not observe a clear association between PHI and mortality when intubation is performed by providers with extended experience. Additional studies are needed to assess whether the observed trend towards better survival is truly a contribution to better outcome or merely a play of chance. Such studies should preferably be adequately powered randomized controlled trials to test the null-hypothesis that prehospital intubation by personnel with ample experience in airway management has no effect on mortality (and possibly other outcomes such as functional recovery) in patients with severe TBI. Herein, the group of healthcare providers who perform intubations, the intubating technique including drugs used to facilitate intubation, as well as the ventilation targets following intubation should be well defined to minimize confounding. Patients with severe TBI form a heterogeneous group. Hence, such a study should either focus on a specific group of patients with well defined characteristics, or specific subgroups need to be defined a priori to allow analyses on whether the effects of prehospital intubation differ depending on patient and injury characteristics.

### Conclusions

Effects of PHI on mortality depend on the EMS-providers’ skill. Prehospital intubation by providers with limited experience is associated with increased mortality, and such providers should not routinely perform PHI in TBI patients. Additional studies are needed to determine the relationship between PHI and mortality when intubation is performed by more experienced personnel.

## Supporting Information

S1 PRISMA Checklist Preferred Reporting Items for Systematic Reviews and Meta-Analyses (PRISMA) checklist. (DOC)

S1 Prospero Protocol Prospective Register of Systematic Reviews (PROSPERO) protocol number CRD42014015506. (PDF)

## Abbreviations

CI: confidence interval
EMS: emergency medical services
GCS: Glasgow Coma Scale
H-AIS: head abbreviated injury score
OR: odds ratio
PHI: prehospital intubation
RCT: randomized controlled trial
TBI: traumatic brain injury
