# Supplementary material for: Experience in Prehospital Endotracheal Intubation Significantly Influences Mortality of Patients with Severe Traumatic Brain Injury: A Systematic Review and Meta-Analysis
Source: PLoS One. 2015 Oct 23;10(10):e0141034. doi: 10.1371/journal.pone.0141034 (PMC4619807; doi:10.1371/journal.pone.0141034)
Supplement: S1 PRISMA Checklist — (DOC) [file pone.0141034.s001.doc]

| **Section/topic** | **#** | **Checklist item** | **Reported on page #** |
| --- | --- | --- | --- |
| **TITLE** | | |  |
| Title | 1 | Identify the report as a systematic review, meta-analysis, or both.  **“Experience in Prehospital Endotracheal Intubation Significantly Influences Mortality of Patients with Severe Traumatic Brain Injury: A Systematic Review and Meta-Analysis”** | 1 |
| **ABSTRACT** | | |  |
| Structured summary | 2 | Provide a structured summary including, as applicable: background; objectives; data sources; study eligibility criteria, participants, and interventions; study appraisal and synthesis methods; results; limitations; conclusions and implications of key findings; systematic review registration number.  **We have used the PLOS abstract format as requested by the PLOS Medicine author guidelines, however the required PRISMA items are included in the abstract** | 2-3 |
| **INTRODUCTION** | | |  |
| Rationale | 3 | Describe the rationale for the review in the context of what is already known.  **“Airway obstruction is common at the accident scene in patients with severe TBI, and resulting hypoxaemia and hypercapnia are known to trigger secondary injuries that adversely affect outcome. Securing the airway is therefore considered a first treatment priority, and prehospital endotracheal intubation – as the "gold standard" of airway management – has often been advocated for comatose trauma patients with a Glasgow Coma Scale (GCS) score of ≤ 8. However, despite theoretical advantages and despite widespread use by emergency medical services (EMS) around the world, there is little scientific evidence to support this practice. In fact, it has even been suggested that prehospital endotracheal intubation may be associated with increased mortality. Hypoxia due to prolonged or failed intubation attempts, increases in intracranial pressure during laryngoscopy, haemodynamic effects of drugs used to facilitate intubation, as well as inappropriate ventilation after endotracheal intubation might all contribute to unfavourable outcomes. In many paramedic based EMS systems, prehospital endotracheal intubation is performed by paramedics who only have basic training in this procedure and infrequently perform intubations in clinical practice. On the other hand, in other EMS systems, endotracheal intubation may be performed by highly trained critical care personnel or emergency physicians. In this context, it is likely that the incidence of adverse events is associated with the level of training and experience in airway management of the provider who performs prehospital intubation (PHI). We therefore hypothesize that PHI by highly trained providers is beneficial, while the same intervention performed by less skilled personnel may be detrimental.”** | 5-6 |
| Objectives | 4 | Provide an explicit statement of questions being addressed with reference to participants, interventions, comparisons, outcomes, and study design (PICOS).  **“We systematically reviewed the available literature and performed a stratified meta-analysis and meta-regression of eligible studies to assess effects of PHI on mortality in patients with severe TBI in the context of the EMS-providers’ experience.”** | 6 |
| **METHODS** | | |  |
| Protocol and registration | 5 | Indicate if a review protocol exists, if and where it can be accessed (e.g., Web address), and, if available, provide registration information including registration number.  **“The study was registered at the International Prospective Register of Systematic Reviews (PROSPERO) with number CRD42014015506 (http://www.crd.york.ac.uk/PROSPERO/display_record.asp?ID=CRD42014015506).”** | 7 |
| Eligibility criteria | 6 | Specify study characteristics (e.g., PICOS, length of follow-up) and report characteristics (e.g., years considered, language, publication status) used as criteria for eligibility, giving rationale.  **“Articles of interest were fully published controlled trials and observational studies comparing PHI versus non-invasive prehospital airway management in patients with suspected or confirmed severe TBI. Severe TBI was defined as a prehospital/admission Glasgow Coma Scale (GCS) ≤ 9 in the presence of a trauma mechanism or findings at physical examination suggestive of head injury, or a Head Abbreviated Injury Score (H-AIS) ≥ 3. Outcome of interest was mortality, and studies that reported or allowed calculation of an effect size were selected. Studies specifically investigating paediatric patients were excluded. Manuscripts reporting other patient populations were considered eligible as long as the data relating to the TBI population could be extracted.**  **A subset of studies was selected for the meta-analysis and meta-regression. Selection criteria were: (1) sufficient quality as described in detail below; (2) the overall study-level EMS-provider experience could be determined; and (3) mortality could be meaningfully compared between intubated and non-intubated patients, i.e., groups were drawn from the same population and are either directly comparable by design with respect to baseline characteristics and injury severity, or adjusted analyses were used to address imbalances between both cohorts.**  **When multiple publications with overlapping data met eligibility criteria for the meta-analysis, or when the same manuscript reported multiple eligible analyses of overlapping data, we used only one of the analyses to avoid duplicate inclusion of patients. In this case, the analysis in which the effect size was estimated with highest precision (i.e., with smallest standard error) was selected. “**  **“We searched PubMed, Embase and Web of Science, without any restrictions, to identify eligible publications.” (I.e. we considered all fully published articles eligible irrespective of publication year or language)** | 7-8 |
| Information sources | 7 | Describe all information sources (e.g., databases with dates of coverage, contact with study authors to identify additional studies) in the search and date last searched.  **“We searched PubMed, Embase and Web of Science, without any restrictions, to identify eligible publications. This search was last updated on July 11th, 2015. (…) Reference lists of pertinent publications were also screened for eligible studies.”** | 8 |
| Search | 8 | Present full electronic search strategy for at least one database, including any limits used, such that it could be repeated.  **“For PubMed, the following search strategy was used: *("intubation, intratracheal"[Mesh] OR "intubation"[Mesh] OR "intubation"[All Fields]) AND ("brain injuries"[Mesh] OR "brain injuries"[All Fields] OR ("brain"[All Fields] AND "injuries"[All Fields]) OR ("head"[All Fields] AND ("injuries"[All Fields] OR "trauma"[All Fields])) OR ("traumatic"[All Fields] AND "brain"[All Fields] AND "injury"[All Fields]) OR ("traumatic brain injury"[All Fields]) OR ("head injury"[All Fields]) OR ("head trauma"[All Fields])) AND ("emergency medical services"[Mesh] OR "prehospital"[All Fields]).* The search terms were adapted accordingly for the other databases.”**  **NB: no limits were used (see item 7: “…without any restrictions”)** | 8 |
| Study selection | 9 | State the process for selecting studies (i.e., screening, eligibility, included in systematic review, and, if applicable, included in the meta-analysis).  **“Two investigators (SMB, PS) independently assessed publications for eligibility by screening abstracts of all identified studies. Full text articles were retrieved for all publications for which the relevance could not be determined based on title and abstract. Disagreements on eligibility were discussed among the investigators, and a third investigator (LAS) was appointed to resolve persisting disagreements.”**  **“A subset of studies was selected for the meta-analysis and meta-regression. Selection criteria were: (1) sufficient quality as described in detail below; (2) the overall study-level EMS-provider experience could be determined; and (3) mortality could be meaningfully compared between intubated and non-intubated patients, i.e., groups were drawn from the same population and are either directly comparable by design with respect to baseline characteristics and injury severity, or adjusted analyses were used to address imbalances between both cohorts.**  **When multiple publications with overlapping data met eligibility criteria for the meta-analysis, or when the same manuscript reported multiple eligible analyses of overlapping data, we used only one of the analyses to avoid duplicate inclusion of patients. In this case, the analysis in which the effect size was estimated with highest precision (i.e., with smallest standard error) was selected.”**  **“A total score of ≥ 7 stars with full score for "comparability" were required as eligibility for the meta-analysis.”**  **See also Table 2 (right-hand side)** | 7-10, Table 2 |
| Data collection process | 10 | Describe method of data extraction from reports (e.g., piloted forms, independently, in duplicate) and any processes for obtaining and confirming data from investigators.  **“Data were extracted by one author (PS) using a standardized data collection sheet, and all data were checked for completeness and accuracy by a second author (SMB). We abstracted information from each included study on: (1) study characteristics, including design, population size, inclusion and exclusion criteria as well as time period and geographical area of patient inclusion; (2) patient characteristics, including age, gender and injury severity; (3) treatments in the intervention and control group; and (4) outcome measures. Studies were classified according to the level of training and experience in performing endotracheal intubations of the group of providers delivering prehospital care. Studies were labelled as “limited experience” if intubation was performed by personnel who usually have basic skills in this technique and who commonly only infrequently perform intubations in routine practice (e.g., emergency medical technicians and paramedics with limited scope of practice). “Extended experience” was selected if intubation was performed by prehospital emergency physicians or nurses/paramedics with an extended scope of practice and training (e.g., specially trained critical care paramedics/nurses). Studies in which the patient population was intubated by a heterogeneous group of providers or in which the level of training could not be ascertained were classified as “indeterminate”. Three reviewers (SMB, LAS, PS) independently assessed and scored the level of experience, and a level was only assigned by unanimous consensus.**  **Seven authors were contacted to obtain additional information, however only three responses were received.”** | 9-10 |
| Data items | 11 | List and define all variables for which data were sought (e.g., PICOS, funding sources) and any assumptions and simplifications made.  **“We abstracted information from each included study on: (1) study characteristics, including design, population size, inclusion and exclusion criteria as well as time period and geographical area of patient inclusion; (2) patient characteristics, including age, gender and injury severity; (3) treatments in the intervention and control group; and (4) outcome measures.”**  **See tables 1, 3, 4 and 5 for all variables, see legend table 5 for an assumption made** | 9, Tables |
| Risk of bias in individual studies | 12 | Describe methods used for assessing risk of bias of individual studies (including specification of whether this was done at the study or outcome level), and how this information is to be used in any data synthesis.  **“Quality assessment was independently performed by two authors (SMB and PS), and a third author (LAS) was consulted in case of disagreement. We used the Newcastle-Ottawa scale to assess the risk of bias of cohort studies. A total of nine stars could be allocated per study for selection of participants, comparability of study groups and assessment of outcome. A total score of ≥ 7 stars with full score for "comparability" were required as eligibility for the meta-analysis.**  **Randomized controlled trials (RCT) were scored using the Cochrane Collaboration’s tool for assessing risk of bias. This tool is used to classify studies as "low", "unclear" and "high" risk of bias. Since blinding of EMS-providers and patients is not possible in studies comparing PHI versus other airway management, the respective item was omitted and studies were classified as "low" risk of bias if no other sources of bias could be identified. Trials with low risk of bias were considered eligible for the meta-analysis. “**  **NB: the Newcastle-Ottawa scale as well as the Cochrane Collaboration’s tool for assessing risk of bias assess bias at the study and outcome level** | 10 |
| Summary measures | 13 | State the principal summary measures (e.g., risk ratio, difference in means).  **“The primary measure of the treatment effect was the odds ratio (OR) of mortality in patients undergoing PHI versus patients who were not intubated in the prehospital setting.”** | 10 |
| Synthesis of results | 14 | Describe the methods of handling data and combining results of studies, if done, including measures of consistency (e.g., I^2^) for each meta-analysis.  **“A meta-analysis of eligible data was performed with STATA 13.0 (StataCorp, Texas). In accordance with our hypothesis that the effect of PHI on mortality differs with EMS-provider experience, and to accommodate for other potential between study heterogeneity, we used a random effects model. Additionally, the analysis was stratified on the EMS-provider's level of experience. Heterogeneity was quantified as the percentage of total variation across studies that is attributable to heterogeneity rather than chance (I^2^-statistic). Random effects meta-regression with EMS-provider experience as trial-level covariate was used to formally assess differences between groups of EMS-providers. “** | 10-11 |

Page 1 of 2

| **Section/topic** | **#** | **Checklist item** | **Reported on page #** |
| --- | --- | --- | --- |
| Risk of bias across studies | 15 | Specify any assessment of risk of bias that may affect the cumulative evidence (e.g., publication bias, selective reporting within studies).  **“We addressed small-study bias in the meta-analysis by plotting the natural logarithm of the odds ratio against its standard error. Funnel plot asymmetry was assessed by Egger’s regression asymmetry test.”** | 11 |
| Additional analyses | 16 | Describe methods of additional analyses (e.g., sensitivity or subgroup analyses, meta-regression), if done, indicating which were pre-specified.  **“… analysis techniques were specified a priori.”**  **“Random effects meta-regression with EMS-provider experience as trial-level covariate was used to formally assess differences between groups of EMS-providers.”**  **“We performed a sensitivity analysis excluding each of the studies one at a time and re-running the analyses without the excluded study.”** | 7, 11, 39 |
| **RESULTS** | | |  |
| Study selection | 17 | Give numbers of studies screened, assessed for eligibility, and included in the review, with reasons for exclusions at each stage, ideally with a flow diagram.  **“The database search provided a total of 1202 articles. Screening of reference lists identified 11 additional articles. A total of 733 articles remained after removal of duplicates/triplicates. Based on review of the abstracts, 614 papers failed to meet the inclusion criteria. The full text of the remaining 119 articles was retrieved and assessed for eligibility. Of those, 95 articles were discarded because they did not meet the inclusion criteria. The remaining 24 studies were included in the systematic review, and six of those studies met the inclusion criteria for the meta-analysis. See figure 1 for the PRISMA flow diagram.”** | 12, Figure 1 |
| Study characteristics | 18 | For each study, present characteristics for which data were extracted (e.g., study size, PICOS, follow-up period) and provide the citations.  **This information is summarized in the text and extensively presented in tables 1, 3, 4 and 5** | 12-31  Tables |
| Risk of bias within studies | 19 | Present data on risk of bias of each study and, if available, any outcome level assessment (see item 12).  **“The Cochrane Collaboration’s tool revealed a low risk of bias for the RCT. The Newcastle-Ottawa Scale rating ranged between four and nine stars for the cohort studies, with a median rating of 7 stars (table 2).”**  **Table 2 shows all details of quality assessment across the different domains** | 18,  Table 2 |
| Results of individual studies | 20 | For all outcomes considered (benefits or harms), present, for each study: (a) simple summary data for each intervention group (b) effect estimates and confidence intervals, ideally with a forest plot.  **“In the 24 studies included in the systematic review, the observed unadjusted OR point estimates ranged between 0.12 and 64.7, while the adjusted estimates ranged between 0.38 and 5.0. In studies where PHI was performed by providers with limited level of training, reported adjusted ORs were between 1.96 and 5.0. In contrast, adjusted ORs were in the range between 0.38 and 0.87 when experienced providers performed intubation. Table 5 reports mortality data of individual studies”**  **Table 5 shows all adjusted and unadjusted mortality data including confidence intervals. Figure 2 is the forest plot of the studies in the meta-analysis** | 31,  Table 5,  Figure 2 |
| Synthesis of results | 21 | Present results of each meta-analysis done, including confidence intervals and measures of consistency.  **“Six analyses including data from 4772 patients met inclusion-criteria for the meta-analysis (table 2). Overall, no significant association was observed between PHI and mortality (OR 1.35, 95% CI 0.78 to 2.33, p=0.279, figure 2). In studies in which intubation was performed by providers with limited experience, PHI was associated with higher odds of mortality (OR 2.33, 95% CI 1.61 to 3.38, p<0.001). In contrast, pooled results in the “extended experience” stratum showed no evidence for higher mortality in patients who were intubated in the prehospital setting (OR 0.75, 95% CI 0.52 to 1.08, p=0.126).” (…) “Substantial heterogeneity was observed between all studies (I^2^=83.3). After adjusting for experience in the meta-regression, residual heterogeneity was negligible (I^2^=10.3%), suggesting that a large portion of the observed heterogeneity can be explained by differences in the level of experience.”**  **Figure 2 is the forest plot of the studies in the meta-analysis** | 39,  Figure 2 |
| Risk of bias across studies | 22 | Present results of any assessment of risk of bias across studies (see Item 15).  **“The funnel plot asymmetry regression test provided no evidence for small study bias (p=0.312).”** | 39 |
| Additional analysis | 23 | Give results of additional analyses, if done (e.g., sensitivity or subgroup analyses, meta-regression [see Item 16]).  **“Meta-regression confirmed that EMS-provider experience is a significant predictor of mortality (p=0.009).”**  **“(…) After adjusting for experience in the meta-regression, residual heterogeneity was negligible (I^2^=10.3%), suggesting that a large portion of the observed heterogeneity can be explained by differences in the level of experience.”**  **“We performed a sensitivity analysis excluding each of the studies one at a time and re-running the analyses without the excluded study. Odds-ratio estimates were of similar magnitude at each exclusion and all conclusions regarding significance remained the same, indicating that none of the included studies has undue influence on the overall results and conclusions (table 6)”** | 39, Table 6 |
| **DISCUSSION** | | |  |
| Summary of evidence | 24 | Summarize the main findings including the strength of evidence for each main outcome; consider their relevance to key groups (e.g., healthcare providers, users, and policy makers).  “**We performed a systematic review and meta-analysis to address effects of PHI on mortality in patients with severe TBI. The main finding is that effects of PHI depend on the level of experience of the EMS-providers who perform the intervention, and that PHI by EMS-personnel with limited experience in performing PHI is associated with increased mortality. When intubation is performed by well-trained personnel, we noted a trend towards improved survival, but the current evidence is insufficient to conclude that PHI by highly trained personnel reduces mortality.”**  **Pages 44 and 45 provide a detailed discussion of the main findings and evidence. We do not specifically discuss the relevance to key groups, because the findings are of high relevance for healthcare providers and policy makers alike.** | 41, 44, 45 |
| Limitations | 25 | Discuss limitations at study and outcome level (e.g., risk of bias), and at review-level (e.g., incomplete retrieval of identified research, reporting bias).  **Limitations are discussed in detail on pages 41 to 44** | 41-44 |
| Conclusions | 26 | Provide a general interpretation of the results in the context of other evidence, and implications for future research.  **“Effects of PHI on mortality depend on the EMS-providers’ skill. Prehospital intubation by providers with limited experience is associated with increased mortality, and such providers should not routinely perform PHI in TBI patients. Additional studies are needed to determine the relationship between PHI and mortality when intubation is performed by more experienced personnel.”** | 45 |
| **FUNDING** | | |  |
| Funding | 27 | Describe sources of funding for the systematic review and other support (e.g., supply of data); role of funders for the systematic review.  **In accordance to the PLOS Medicine instructions to authors, we do not report Funding in the manuscript (“Do not include funding sources in the Acknowledgments or anywhere else in the manuscript file. Funding information should only be entered in the financial disclosure section of the online submission system.”)** | NR |
