# Supplementary material for: Experience in Prehospital Endotracheal Intubation Significantly Influences Mortality of Patients with Severe Traumatic Brain Injury: A Systematic Review and Meta-Analysis
Source: PLoS One. 2015 Oct 23;10(10):e0141034. doi: 10.1371/journal.pone.0141034 (PMC4619807; doi:10.1371/journal.pone.0141034)
Supplement: S1 Prospero Protocol — (PDF) [file pone.0141034.s002.pdf]

PROSPERO International prospective register of systematic reviews

Review title and timescale

1 Review title
Give the working title of the review. This must be in English. Ideally it should state succinctly the interventions or exposures being reviewed and the associated health or social problem being addressed in the review.
Effects of prehospital tracheal intubation on survival in patients with traumatic brain injury

2 Original language title
For reviews in languages other than English, this field should be used to enter the title in the language of the review. This will be displayed together with the English language title.

3 Anticipated or actual start date
Give the date when the systematic review commenced, or is expected to commence.
01/07/2014

4 Anticipated completion date
Give the date by which the review is expected to be completed.
30/01/2015

5 Stage of review at time of this submission
Indicate the stage of progress of the review by ticking the relevant boxes. Reviews that have progressed beyond the point of completing data extraction at the time of initial registration are not eligible for inclusion in PROSPERO. This field should be updated when any amendments are made to a published record.

The review has not yet started ×

Review stage	Started	Completed
Preliminary searches	Yes	Yes
Piloting of the study selection process	Yes	Yes
Formal screening of search results against eligibility criteria	Yes	Yes
Data extraction	Yes	Yes
Risk of bias (quality) assessment	Yes	Yes
Data analysis	Yes	Yes

Provide any other relevant information about the stage of the review here.

Review team details

6 Named contact
The named contact acts as the guarantor for the accuracy of the information presented in the register record.
Dr Schober

7 Named contact email
Enter the electronic mail address of the named contact.
p.schober@vumc.nl

8 Named contact address
Enter the full postal address for the named contact.
VU University Medical Center Department of Anaesthesiology (ZH 6F 028) De Boelelaan 1117 1081 HV Amsterdam The Netherlands

9 Named contact phone number
Enter the telephone number for the named contact, including international dialing code.
+31 (0)20 444 3138

10 Organisational affiliation of the review
Full title of the organisational affiliations for this review, and website address if available. This field may be completed as 'None' if the review is not affiliated to any organisation.

VU University Medical Center

Website address:

11 Review team members and their organisational affiliations

Give the title, first name and last name of all members of the team working directly on the review. Give the organisational affiliations of each member of the review team.

Title	First name	Last name	Affiliation
Dr	Patrick	Schober	VU University Medical Center
Dr	Sebastiaan M.	Bossers	VU University Medical Center
Dr	Lothar A.	Schwarte	VU University Medical Center

12 Funding sources/sponsors

Give details of the individuals, organizations, groups or other legal entities who take responsibility for initiating, managing, sponsoring and/or financing the review. Any unique identification numbers assigned to the review by the individuals or bodies listed should be included.

Department of Anaesthesiology, VU University Medical Center. Dutch Brain Foundation.

13 Conflicts of interest

List any conditions that could lead to actual or perceived undue influence on judgements concerning the main topic investigated in the review.

Are there any actual or potential conflicts of interest?

None known

14 Collaborators

Give the name, affiliation and role of any individuals or organisations who are working on the review but who are not listed as review team members.

Title	First name	Last name	Organisation details
Professor	Christa	Boer	VU University Medical Center
Professor	Stephan A.	Loer	VU University Medical Center
Professor	Jos W.R.	Twisk	VU University Medical Center

Review methods

15 Review question(s)

State the question(s) to be addressed / review objectives. Please complete a separate box for each question.

What is the relationship between prehospital endotracheal intubation and mortality in patients with severe traumatic brain injury?

Does the relationship between prehospital endotracheal intubation and mortality in patients with severe traumatic brain injury depend on the level of training/experience of the healthcare provider who performs the intervention?

16 Searches

Give details of the sources to be searched, and any restrictions (e.g. language or publication period). The full search strategy is not required, but may be supplied as a link or attachment.

PubMed, EMBASE and Web of Knowledge are searched without any language or time period restrictions. The search strategy uses MeSH terms as well as text search in all fields to identify all studies that report data from patients with head trauma / traumatic brain injury who are intubated in the prehospital setting.

17 URL to search strategy

If you have one, give the link to your search strategy here. Alternatively you can e-mail this to PROSPERO and we will store and link to it.

I give permission for this file to be made publicly available

Yes

18 Condition or domain being studied

Give a short description of the disease, condition or healthcare domain being studied. This could include health and wellbeing outcomes.
Disease: severe traumatic brain injury. Outcome: mortality.

19 Participants/population
Give summary criteria for the participants or populations being studied by the review. The preferred format includes details of both inclusion and exclusion criteria.
Inclusion criteria: Publications of interest are controlled trials and observational studies comparing prehospital endotracheal intubation versus non-invasive prehospital airway management in patients with suspected or confirmed severe traumatic brain injury. Severe traumatic brain injury is defined as either a prehospital/admission Glasgow Coma Scale (GCS) = 3. Exclusion criteria: Studies specifically investigating the pediatric patient population as well as studies which are only reported as conference abstracts but have not fully been published in peer-reviewed journals are excluded.

20 Intervention(s), exposure(s)
Give full and clear descriptions of the nature of the interventions or the exposures to be reviewed
Endotracheal intubation with (rapid sequence intubation) and without medications in the prehospital setting

21 Comparator(s)/control
Where relevant, give details of the alternatives against which the main subject/topic of the review will be compared (e.g. another intervention or a non-exposed control group).
No endotracheal intubation in the prehospital setting

22 Types of study to be included initially
Give details of the study designs to be included in the review. If there are no restrictions on the types of study design eligible for inclusion, this should be stated.
Controlled trials and observational studies

23 Context
Give summary details of the setting and other relevant characteristics which help define the inclusion or exclusion criteria.

24 Primary outcome(s)
Give the most important outcomes.
Mortality during hospitalisation or at other well defined time points (e.g., 30 days after trauma)

Give information on timing and effect measures, as appropriate.

25 Secondary outcomes
List any additional outcomes that will be addressed. If there are no secondary outcomes enter None.
None

Give information on timing and effect measures, as appropriate.

26 Data extraction, (selection and coding)
Give the procedure for selecting studies for the review and extracting data, including the number of researchers involved and how discrepancies will be resolved. List the data to be extracted.
Study selection: Two investigators (SB, PS) independently assessed publications for eligibility by screening titles and abstracts of all identified studies. Full text articles were retrieved for all eligible publications as well as for all publications for which the relevance could not be determined based on title and abstract alone. Disagreements on eligibility were discussed among the investigators, and a third investigator (LS) had been appointed to resolve persisting disagreements. Data extraction: Data were extracted from eligible studies by one author (PS) using a standardized data collection sheet, and all data were checked for completeness and accuracy by a second author (SB). We abstracted information from each included study on: (1) study characteristics, including study design, population size, reported inclusion and exclusion criteria as well as time period and geographical area of patient inclusion; (2) patient characteristics, including age, gender and severity of TBI; (3) type of interventions, including invasive and non-invasive airway management techniques; and (4) outcome measures. Additionally, studies were dichotomized according to the level of training and experience in performing endotracheal intubations of the group of providers delivering prehospital health care. Studies were labeled as “limited/moderate experience” if intubation was performed by personnel who usually only infrequently perform intubations in routine practice (e.g. emergency medical

technicians and basic/intermediate level paramedics). “Intermediate/high experience” was selected if intubation was performed by paramedics/nurses who had reportedly been specially and thoroughly trained in this procedure or by emergency physicians with regular intubation experience. Three reviewers (SB, LS, PS) independently assessed and scored the level of experience, and a level was only assigned by unanimous decision.

27 Risk of bias (quality) assessment

State whether and how risk of bias will be assessed, how the quality of individual studies will be assessed, and whether and how this will influence the planned synthesis.

Quality assessment will be independently performed by two authors (SB and PS), and a third author (LS) will be consulted in case of disagreement. Randomized controlled trials will be assessed using the Cochrane Collaboration’s tool for assessing risk of bias. This tool is used to classify studies as "low", "unclear" and "high" risk of bias depending on the adequacy of sequence generation, allocation concealment, blinding, handling of incomplete outcome data, selective outcome reporting, and assessment of other sources of bias. Since blinding of healthcare providers and patients is not possible in studies comparing endotracheal intubation versus other airway management, this item is omitted and studies are classified as "low" risk of bias if no other relevant sources of bias can be identified. The Newcastle-Ottawa scale will be used to assess the quality and risk of bias of observational studies. A total of nine stars can be allocated per study for selection of participants (maximum four stars), comparability of study groups (maximum of two stars) and exposure or outcome, respectively (maximum three stars).

28 Strategy for data synthesis

Give the planned general approach to be used, for example whether the data to be used will be aggregate or at the level of individual participants, and whether a quantitative or narrative (descriptive) synthesis is planned. Where appropriate a brief outline of analytic approach should be given.

A narrative synthesis of the findings from the included studies will be provided. A subset of studies of sufficient quality (see below) and in which both groups of patients (i.e., intubated versus non-intubated) are directly comparable with respect to baseline characteristics and injury severity are selected for a quantitative analysis (meta-analysis and meta-regression). Randomized controlled trials with low risk of bias are considered eligible for the meta-analysis. For observational studies, a total Newcastle-Ottawa score of >= 7 stars and 1 star for selection of the non-exposed cohort and 2 stars for "comparability" are required as eligibility for the meta-analysis.

29 Analysis of subgroups or subsets

Give any planned exploration of subgroups or subsets within the review. ‘None planned’ is a valid response if no subgroup analyses are planned.

The meta-analysis will be stratified according to the experience of the healthcare providers who perform the intubation (“limited/moderate experience” versus “intermediate/high experience” as described above). Meta regression with provider experience as covariate will be used to formally assess differences between experience groups.

Review general information

30 Type of review

Select the type of review from the drop down list.

Intervention

31 Language

Select the language(s) in which the review is being written and will be made available, from the drop down list. Use the control key to select more than one language.

English

Will a summary/abstract be made available in English?

Yes

32 Country

Select the country in which the review is being carried out from the drop down list. For multi-national collaborations select all the countries involved. Use the control key to select more than one country.

Netherlands

33 Other registration details

Give the name of any organisation where the systematic review title or protocol is registered together with any unique identification number assigned. If extracted data will be stored and made available through a repository such as the Systematic Review Data Repository (SRDR), details and a link should be included here.

34 Reference and/or URL for published protocol
Give the citation for the published protocol, if there is one.
Give the link to the published protocol, if there is one. This may be to an external site or to a protocol deposited with CRD in pdf format.

I give permission for this file to be made publicly available
Yes

35 Dissemination plans
Give brief details of plans for communicating essential messages from the review to the appropriate audiences.
Do you intend to publish the review on completion?
Yes

36 Keywords
Give words or phrases that best describe the review. (One word per box, create a new box for each term)
Traumatic brain injury

Endotracheal intubation

Prehospital Treatment

Emergency Medical Services

37 Details of any existing review of the same topic by the same authors
Give details of earlier versions of the systematic review if an update of an existing review is being registered, including full bibliographic reference if possible.

38 Current review status
Review status should be updated when the review is completed and when it is published.
Completed but not published

39 Any additional information
Provide any further information the review team consider relevant to the registration of the review.

40 Details of final report/publication(s)
This field should be left empty until details of the completed review are available.
Give the full citation for the final report or publication of the systematic review.
Give the URL where available.
